# Clinical presentation and management of acromegaly in elderly patients

**DOI:** 10.1007/s42000-020-00235-5

**Published:** 2020-08-25

**Authors:** Filippo Ceccato, Mattia Barbot, Laura Lizzul, Angela Cuccarollo, Elisa Selmin, Isabella Merante Boschin, Andrea Daniele, Alois Saller, Gianluca Occhi, Daniela Regazzo, Carla Scaroni

**Affiliations:** 1 Endocrinology Unit, Department of Medicine DIMED, University Hospital of Padova, Via Ospedale Civile, 105-35128 Padova, Italy; 2 Department of Neurosciences DNS, University of Padova, Padova, Italy; 3 Department of Surgical, Oncological and Gastroenterological Sciences DiSCOG, University of Padova, Padova, Italy; 4 Internal Medicine, Department of Medicine DIMED, University Hospital of Padova, Padova, Italy; 5 Department of Biology, University of Padova, Padova, Italy

**Keywords:** Acromegaly, Diagnosis, Medical treatment, Aging

## Abstract

**Background and aim:**

Acromegaly is a rare disease with a peak of incidence in early adulthood. However, enhanced awareness of this disease, combined with wide availability of magnetic resonance imaging (MRI), has increased the diagnosis of forms with mild presentation, especially in elderly patients. Moreover, due to increased life expectancy and proactive individualized treatment, patients with early-onset acromegaly are today aging. The aim of our study was to describe our cohort of elderly patients with acromegaly.

**Materials and methods:**

This is a cross-sectional retrospective study of 96 outpatients. Clinical, endocrine, treatment, and follow-up data were collected using the electronic database of the University Hospital of Padova, Italy.

**Results:**

We diagnosed acromegaly in 13 patients, aged ≥65 years, presenting with relatively small adenomas and low IGF-1 secretion. Among them, 11 patients were initially treated with medical therapy and half normalized hormonal levels after 6 months without undergoing neurosurgery (TNS). Remission was achieved after TNS in three out of four patients (primary TNS in two); ten patients presented controlled acromegaly at the last visit. Acromegaly-related comorbidities (colon polyps, thyroid cancer, adrenal incidentaloma, hypertension, and bone disease) were more prevalent in patients who had an early diagnosis (31 patients, characterized by a longer follow-up of 24 years) than in those diagnosed aged ≥65 years (5 years of follow-up).

**Conclusions:**

Elderly acromegalic patients are not uncommon. Primary medical therapy is a reasonable option and is effectively used, while the rate of surgical success is not reduced. A careful cost-benefit balance is suggested. Disease-specific comorbidities are more prevalent in acromegalic patients with a longer follow-up rather than in those diagnosed aged ≥65 years.

## Introduction

Acromegaly is a rare disease, usually due to a pituitary adenoma, characterized by increased GH and IGF-1 levels.[1, 2] It is usually diagnosed in young adults (between 35 and 45 years), and only a few cases are reported as a new diagnosis in elderly subjects (≥ 65 years).[3, 4] Some of the clinical features of acromegaly (especially acral and facial modifications) or GH-related complications (e.g., impaired glucose metabolism, arterial hypertension, sleep apnea, goiter, and colon polyps[5–8]) may be misinterpreted as a consequence of aging, the patient, as a result, not achieving a correct and prompt diagnosis. On the other hand, new-onset acromegaly is not uncommon in the geriatric population, and increased awareness about this disease among the medical community[9] will help reduce delayed diagnosis. Moreover, the widespread use of magnetic resonance imaging (MRI) has led to increased identification of pituitary incidentalomas, thus raising the number of screening procedures for GH excess in patients without an overt clinical picture.[10]

In cases of active or untreated acromegaly, life expectancy is reduced due to cardiovascular, respiratory, metabolic, and neoplastic comorbidities.[11, 12] However, optimal control of the disease with surgical and/or medical treatments (somatostatin analogs, SSA; dopamine receptor agonists, DA; and growth hormone-receptor antagonists, GH-RA) has modified the natural history of the disease over the last few decades and many acromegalic patients may survive until old age.[13]

The increase in life expectancy in European countries,[14] especially in the Italian population,[15] is responsible for the growing number of elderly patients with morbidities, including pituitary adenomas. Few studies, however, have reported the characteristics of pituitary adenomas in the elderly,[16–18] especially with regard to acromegaly.[3, 4, 19, 20] Moreover, due to tailored treatment, acromegalic patients are today aging. Therefore, we now observe two groups of elderly subjects with acromegaly: those with a new diagnosis after 65 years of age and those with early-onset acromegaly who have grown old over time.[21]

The aims of this study were thus to compare clinical characteristics, GH-related comorbidities, and different therapeutic approaches to treatment of elderly patients with a new diagnosis of acromegaly or those who have become elderly after adult-onset acromegaly.

## Materials and methods

We conducted a cross-sectional retrospective study. Among 120 adult patients with acromegaly followed up at our Endocrinology Unit at the University Hospital of Padova, Italy, we considered 96 individuals with complete information (biochemical and radiological evaluation at diagnosis) and clinical follow-up (at least 12 months after diagnosis or 6 months after surgery).

In all patients, biochemical diagnosis was defined as lack of GH suppression (< 1 μg/l or 0.4 μg/l, according to the Endocrine Society guidelines in use at the time of diagnosis) during a standard oral glucose tolerance test (OGTT) with 75 g of glucose and increased IGF-1 levels, normalized for age and gender.[22] The same analyses were used to confirm remission after transnasal-sphenoidal surgery (TNS) or radiotherapy; random GH < 1 μg/l and IGF-1 below the upper limit of normality (ULN, normalized for age and gender) allowed us to define good management of the disease during medical treatment (only IGF-1 in patients treated with GH-RA).[6]

We evaluated the medical records of the selected patients with the aim of identifying the different therapeutic approaches used, remission after neurosurgery and/or radiotherapy, and disease management with medical therapy. All patients enrolled in this study underwent periodical clinical evaluation (at least once per year) in the setting of the routine ambulatory practice. Clinical data were reported in the web-based database of the University Hospital of Padova, used as an electronic case report/record form. Regarding comorbidities, we considered glucose metabolism, goiter, differentiated thyroid carcinoma (DTC), colonic polyposis, hepatic steatosis, benign hepatic nodules (hemangioma or cyst), cholelithiasis, adrenal incidentalomas, arterial hypertension, left ventricular hypertrophy, intracranial aneurysms, and bone disease (radiological evidence of osteoporosis or vertebral fractures). The detection of acromegaly-related complications is strictly dependent on the methods used to investigate them; however, in our clinical practice, we perform a scheduled follow-up accepted worldwide in referral centers,[23] including a thyroid ultrasound (at acromegaly diagnosis, with a cytological examination of the suspicious nodes if needed, and then every 2 years).

Patients were divided into three categories, namely, group 1: subjects <65 years at diagnosis and at the last clinical evaluation; group 2: subjects <65 years at diagnosis and ≥ 65 years at last follow-up; and group 3: elderly patients aged ≥65 years at diagnosis.

Magnetic resonance (MR) 1.5 T scanning was performed with a standard quadrature head coil (Achieva; Philips Medical Systems, Best, the Netherlands); all patients underwent T1-and T2-weighted gadolinium-enhanced MR. Pituitary tumors were classified by maximal diameter as microadenomas (< 10 mm) or macroadenomas (≥ 10 mm). Patients were treated with SSA (octreotide LAR or lanreotide autogel), DA (cabergoline), or GH-RA (pegvisomant, allowed in Italy after surgical failure).

Pituitary deficiencies were diagnosed as described elsewhere; briefly, GH deficiency was confirmed by a GHRH + arginine stimulation test with BMI-based cut-off.[24] Central adrenal insufficiency was evaluated with basal serum cortisol or after a short Synacthen test when appropriate.[25] Diagnosis of other hormone deficiencies (central hypothyroidism or hypogonadism) was based on low or inappropriately normal pituitary hormone levels with lower than normal serum fT4 or gonadal steroid hormones.

The Ethics Committee of Padova University Hospital approved the study protocol and all patients gave written informed consent.

We calculated proportions and rates or medians and interquartile ranges (IQR) for nonparametric variables. Groups were compared with the Pearson Chi-square test and the Mann-Whitney test for quantitative variables.

To take into account multiple comparisons, the raw *p* values were adjusted with the Bonferroni method for multiple comparisons. The SPSS 17 software package (SPSS, Inc., Chicago, IL, USA) was used for all analyses. The significance level was set at *p* < 0.05 for all tests.

## Results

### Diagnosis of acromegaly after vs. before 65 years of age

Acromegaly was diagnosed in patients after 65 years of age in 13 cases (14% of the whole cohort), described in Table 1: mandibular and acral overgrowth, the skeletal manifestations of acromegaly, were not the main clinical presentation of acromegaly in seven of the 13 subjects. In Table 2, we report clinical and radiological data: patients with a diagnosis of acromegaly after 65 years of age presented with lower IGF-1 levels (with similar levels considering ULN) and with smaller adenomas than subjects with early diagnosis (< 65 years of age).

**Table 1:** Patients aged ≥65 years at diagnosis

Case, gender	Age at diagnosis	Signs and symptoms at diagnosis that first arouse suspicion of acromegaly	Basal GH (μg/L) nadir OGTT	Basal IGF-1 (μg/L)	Basal IGF-1 (ULN)	Adenoma at diagnosis (mm)	Surgery/outcome	Medical treatment last control	Hypopituitarism	Last random GH (μg/L)	Last IGF-1 (μg/L)	Last IGF-1 (ULN)
1, M, 72	65 years	Erectile dysfunction	6.4	447	2.14	9	Yes/remission	n.a.	No	0.73	200	0.97
2, M, 70	65 years	Headache ➔ pituitary incidentaloma	5.9	600	2.87	10	Yes/remission	n.a.	No	0.11	221	0.93
3, F, 80	66 years	Papillary thyroid carcinoma, obstructive sleep apnea syndrome	9.6	695	2.88	23	No	SSA + CAB	?No	4.58	99	0.45
4, F, 72	67 years	Carpal tunnel syndrome, mandibular overgrowth	4.76	752	3.12	11	Yes/remission	n.a.	TSH/ACTH	1.4	209	0.88
5, M, 72	67 years	Mandibular and acral overgrowth, thyroid goiter	1.78	357	1.7	12	?No	SSA	No	4.06	205	0.99
6, F, 75	68 years	Profuse sweating	23	612	2.93	9	Yes/persistence	SSA + GH-RA	No	0.78	220	0.93
7, F, 78	68 years	Papillary thyroid carcinoma, osteoporosis	6.88	1060	4.4	10	No	SSA	No	0.32	161	0.74
8, F, 75	68 years	Carpal tunnel syndrome, acral overgrowth	2.57	314	1.3	10	No	SSA + CAB	No	0.68	152	0.64
9, F, 75	69 years	Obstructive sleep apnea syndrome, mandibular and acral overgrowth	6.36	575	2.39	11	No	SSA	No	0.31	212	0.89
10, F, 82	74 years	Transient ischemic attack ➔ pituitary incidentaloma	25.8	686	2.89	12	No	SSA + CAB	No	9.69	388	1.77
11, F, 94	75 years	Mandibular and acral overgrowth	4	636	2.68	9	No	SSA	No	2.7	233	1.98
12, F, 83	78 years	Mandibular and acral overgrowth	18.7	823	3.76	15	No	SSA	No	2.01	254	1.16
13, M, 88	78 years	Meningioma ➔ pituitary incidentaloma	25.2	570	2.82	9	No	SSA	No	1.96	158	0.78

**Table 2:** Clinical characteristics of patients at diagnosis (data are reported as median and IQR)

	All cohort (*n* = 96)	< 65 years (*n* = 83)	≥ 65 years (*n* = 13)	*P*
Gender	40 male/56 female	36 male/47 female	4 male/9 female	0.293^a^
GH (μg/L) nadir after OGTT	6.9 (2.6–17.5)	6.8 (3.6–21.9)	6.9 (2.1–16)	0.587^b^
IGF-1 (μg/L)	715 (574–978)	748 (602–996)	600 (447–752)	0.022^b^
IGF-1/normal IGF-1 (ULN)	2.91 (2.26–3.6)	3 (2.25–6.63)	2.87 (2.14–3.12)	0.371^b^
Micro−/macroadenoma	20/60 (25%/75%)	16/52 (24%/76%)	4/8 (33%/67%)	0.465^a^
Maximum diameter of adenoma	15 (10–20)	15 (11–20)	11 (9–13)	0.024^b^

^a^Pearson Chi-square test

^b^Mann-Whitney test

**Fig. 1:**
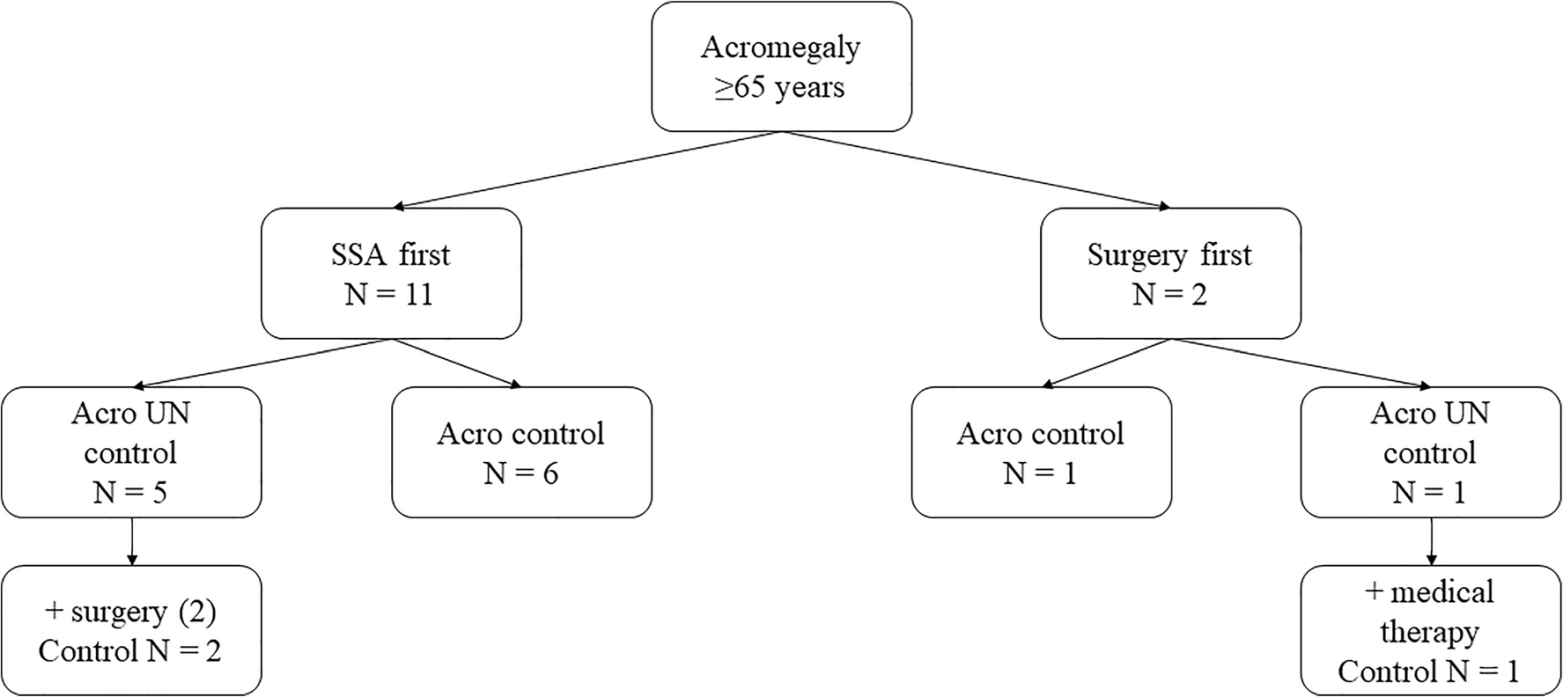
Acromegaly treatment in patients aged >65 years. SSA: somatostatin analogs; control: patients with normalized GH and IGF-1 levels, UN control: patients with increased GH or IGF-1 levels

As reported in Fig. 1, patients older than 65 years at diagnosis (group 3) were treated with SSA or surgery. Two patients underwent neurosurgery as first-line treatment: one achieved remission after TNS and the other with adjuvant medical therapy. On the other hand, 11 patients were treated with primary medical therapy: six achieved normalization of hormonal levels after 6 months of treatment. On the whole, four of the 13 patients underwent TNS (two after medical treatment), with a final remission rate of 75%. Acromegaly was controlled in ten of the 13 elderly patients at the last clinical evaluation, with SSA alone (six cases), or combined with DA or GH-RA (as depicted in Table 1).

Patients younger than 65 years at diagnosis (groups 1 and 2) represented 86% of the population considered in this study; 64% (53/83) underwent neurosurgery as first-line treatment. Among them, seven patients experienced repeated surgery, achieving remission in one case. Nineteen patients underwent radiotherapy. At the last visit, 13 were in remission, while six still needed adjuvant medical therapy. At the last follow-up, 69 out of 83 patients with acromegaly diagnosed before 65 years (83%) showed normalization of GH and IGF-1 levels.

As reported in Table 3, we compared the management of the disease in patients under or over 65 years at diagnosis. Overall, 75/96 underwent neurosurgery, 71 of them were aged <65 years. Overall, TNS was offered especially to group 1 (48 out of 52 cases, 92%) rather than group 2 (23 out of 31 cases, 74%) or group 3 (4 out of 13 subjects, 31%, *p* < 0.001). Subjects in group 3 achieved a higher remission rate after TNS than younger acromegalic patients: 3/4 (75%) vs. 44/71 (62%, *p* = 0.016). Medical treatment was often considered: 91% (87/96) of patients were treated with medical therapy for at least 6 months. We did not find any significant difference in the treatment schedule or in the treatment response between the three groups. At the last clinical evaluation, 40/96 patients were still on medical therapy, without a different approach based on age at diagnosis.

**Table 3:** Different acromegaly treatments according to age of onset

	< 65 years (*n* = 83)	≥ 65 years (*n* = 13)	*p*
Surgery	71 (86%)	4 (31%)	< 0.001^*^
Medical treatment	75 (90%)	12 (92%)	0.823^*^
Radiotherapy	19 (23%)	0 (0%)	0.045^*^
Primary medical therapy and secondary surgery	23 (28%)	2 (15%)	0.282^*^

^a^Pearson Chi-square test

### Elderly patients with acromegaly

Thirty-one patients (19 female) met the inclusion criteria of group 2 (diagnosis of acromegaly before 65 years and age at the last clinical record ≥65 years). Median age at diagnosis was 50 years (IQR 41–57) and median age at last follow-up was 71 years (IQR 68–76, similarly to group 3 median 73 years, IQR 70–81, *p* = 0.601), without gender differences. At diagnosis, 63% presented with a macroadenoma; surgery was performed in 23 patients, and 13 were radio-treated; 19 are still on medical treatment (17 SSA, two cabergoline, two GH-RA alone, and two combined GH-RA + SSA). Comparing this cohort of patients with young acromegalic subjects (group 1), TNS was preferentially proposed to younger patients (48/52 vs. 23/31, *p* = 0.023), radiotherapy was performed more in the second group (6/52 vs. 13/31, *p* = 0.002), and the choice of medical treatment was similar (*p* = 0.993).

**Fig. 2:**
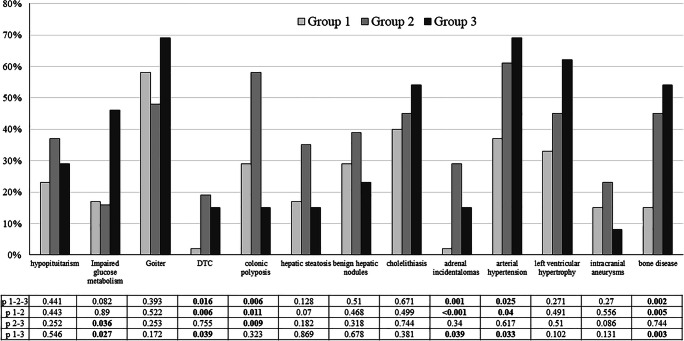
Comorbidities according to age group. Group 1: age at diagnosis and at last follow-up <65 years; group 2: age at diagnosis <65 years and at last follow-up ≥65 years; group 3: age at diagnosis ≥65 years. P 1–2-3: group 1 vs. 2 vs. 3 (with Bonferroni correction); p 1–2: group 1 vs. 2; p 2–3 group 2 vs. group 3

Median follow-up was longer in group 2 (24 years, IQR 17–31) than in group 1 (10 years, IQR 6–14, *p* < 0.001) and in group 3 (5 years, IQR 3–10, *p* = 0.024 vs. group 1 and *p* < 0.001 vs. group 3). As reported in Fig. 2, acromegaly-related comorbidities were more prevalent in elderly acromegalic patients with early diagnosis (group 2, characterized by the longer follow-up), especially DTC, colonic polyps, adrenal incidentalomas, arterial hypertension, and bone disease. In contrast, impaired glucose metabolism was more prevalent when acromegaly was discovered after 65 years of age.

## Discussion

Acromegaly is a rare disease, and despite its insidious onset, it is often diagnosed in early-adult patients.[2] Nevertheless, greater awareness of the disease and increased availability of MRI in the last few decades have led to an increase in new diagnoses of acromegaly. Moreover, life expectancy among the general population is longer than a few decades ago[14]; therefore, new acromegaly diagnoses after the age of 65 years are not uncommon. However, to the best of our knowledge, there is still scant knowledge regarding the management of acromegaly in elderly subjects.

Acromegaly has become quite common among the elderly. In our series, 14% of new diagnoses were performed in patients over 65 years of age (up to 78 years), and 46% of patients are above 65 years at the last visit. This unexpectedly high prevalence of a rare disease in elderly subjects is probably due to the fact that our center is a European referral center for rare endocrine diseases (Endo-ERN). However, some patients were referred to our center for several different reasons, often without the initial recognition of acromegaly. Indeed, two patients were diagnosed during the work-up with a pituitary incidentaloma, and two were referred to the Endocrine Unit for thyroid cancer. In such cases, though the features of acromegaly were present, it is likely that were not so striking as to suggest a diagnosis of GH excess: we can speculate that mild acral modifications accompanied by metabolic and cardiovascular complications could be misinterpreted and sometimes considered as age-related features.

The clinical overlap between acromegaly in the elderly and physiological aging could be partly explained by a reduced rate of IGF-1 secretion, as was observed in our cohort. It has recently been reported that older patients present with lower basal IGF-1 levels as well as smaller pituitary adenomas[26, 27]; nevertheless, in our cohort, all patients presented invariably with increased basal GH, thus excluding the novel entity called micromegaly.[26] Regarding IGF-1 levels, the absolute values of IGF-I were lower in group 3; however, it must be noted that GH secretion decreases with age in both normal and acromegalic individuals.[28] Therefore, ULN of IGF-1 levels did not differ between the groups when the levels were adequately adjusted for gender and age. With regard to tumor size, the cohort diagnosed at an older age showed a tendency to present with smaller adenomas than the younger diagnosed group. On the one hand, the diagnosis of acromegaly has evolved over the years from overt clinical manifestations (as viewed in subjects with advanced disease, thus exhibiting unambiguous signs and symptoms) to a milder phenotype thanks to the widespread use of MRI and ultrasensitive GH or IGF-1 assays. On the other hand, we can hypothesize the existence of two distinct phenotypes of acromegaly: young subjects with macroadenomas and overt phenotype (with poor response to medical treatment and to genetic variants) and older patients harboring microadenomas, characterized by lower GH secretion leading to a milder phenotype.

Neurosurgery is considered the first-line treatment, especially for microadenomas or non-invasive macroadenomas.[6, 29] Surgical expertise is one of the main determinants of the outcome, achieving satisfactory remission rate only in referral centers.[30, 31]

In our series, elderly patients underwent TNS less frequently than younger subjects, even if presenting with smaller tumors that usually have better chances for curative surgery. In accordance with this, older subjects showed a better response to surgery, achieving a remission rate close to that recently reported in a large series of elderly patients with pituitary adenoma.[18] Sub-analyzing the four patients who underwent TNS (two as primary treatment, two after the failure of SSA treatment), they were all between 65 and 68 years and presented a good performance status. However, our data are not consistent enough to suggest surgery as first-line treatment for older or more fragile patients. Primary medical treatment was offered to the majority of our geriatric patients (11/13 cases), achieving IGF-1 and GH normalization in 55% of cases, similar to that reported in a meta-analyses[32] and confirming that medical treatment, before or instead of surgery, is a reasonable choice.[13] Moreover, the risk of pituitary failure induced by medical treatment is reduced,[33] unlike for surgery or radiotherapy. In such a scenario, it must also be borne in mind that medical therapy constitutes life-long treatment with an estimated cost of up to 10,000 €/patient/year[34] and that it raises the risk of glucose metabolism impairment,[5] which is also age-dependent in patients without acromegaly. Hence, the best choice of treatment for elderly acromegalic patients is a matter of debate: on the one hand, they are often poor surgical candidates due to age- and acromegaly-related comorbidities; on the other, a careful cost-benefit analysis is strongly advised. A dedicated multicenter prospective study should be conducted to better clarify the risks and the benefits of the surgical choice in elderly acromegalic patients.

As expected, older patients were not radio-treated. This clinical attitude could be secondary to the evidence that it takes years to reduce GH secretion[35] in combination with the risk of new-onset hypopituitarism[36, 37] and parenchymal brain injuries.[38] Moreover, the French Acromegaly Registry has described a trend toward ever fewer recommendations of radiotherapy over the past three decades (< 20% of all patients).[8]

Regarding comorbidities, it must be noted that the duration of follow-up was different in the three cohorts of subjects, being higher in those with early onset of acromegaly and an age of >65 years at the last follow-up, as expected. The higher rate of comorbidities observed in elderly acromegalic patients with an early diagnosis (group 2, characterized by a longer duration of disease) than in those with acromegaly diagnosed after 65 years can be related to the prolonged exposure to GH and IGF-1 and to a late diagnosis, added to physiological aging. Moreover, the reduced GH secretion in patients diagnosed at >65 years of age could also play a role regarding the lower incidence of acromegaly-related complications. Increased cancer risk in acromegaly is a matter of debate[39]: a close relationship between the oncological risk and the endocrine activity of acromegaly (GH, IGF-1 and insulin) has yet to be defined,[40] and age, genetic, and epigenetic factors are involved.[41, 42] As previously reported, we observed in older patients a higher incidence of thyroid cancer,[43, 44] colonic polyps,[45] and adrenal incidentalomas.[46] Regarding cardiovascular comorbidities, the increased prevalence of hypertension with aging could also be attributed to its usual upward trend in elderly subjects[47] rather than the high prevalence of hypertension in acromegaly.[11]

Besides strengths, our work also presents several limitations. First of all, there is the intrinsic retrospective nature of a cross-sectional study without a control group. Moreover, it is a single-center study with a relatively small sample size due to the rarity of the disease. Second, the decisions on the best treatment options were not always taken by the same team over time, since patients often moved to our unit after being treated in other centers.

To conclude, acromegaly is not uncommon in elderly subjects, and its diagnosis might be misinterpreted in those aged over 65 years due to the overlap of cardiovascular comorbidities with physiological aging and a mild phenotype. Although primary medical therapy is a reasonable choice and is effectively used, the rate of surgical success is not reduced in seniors. Any treatment that is offered to selected patients must be decided upon after a careful cost-benefit analysis. In our long-term evaluation, we observed how the prevalence of some of the most common acromegaly-related comorbidities is higher in elderly patients with a longer follow-up. Further studies are needed to establish what role is played by aging and disease duration in the development of the main comorbidities of acromegaly.

## Data Availability

Data are available on request due to local (academic) restrictions.
